# Micro-drive and headgear for chronic implant and recovery of optoelectronic probes

**DOI:** 10.1038/s41598-017-03340-5

**Published:** 2017-06-05

**Authors:** Jinho Chung, Farnaz Sharif, Dajung Jung, Soyoun Kim, Sebastien Royer

**Affiliations:** 1 Center for Functional Connectomics, Korea Institute of Science and Technology, Seoul, 136-791 Republic of Korea; 2 Department of Biological Sciences, Korea Advanced Institute of Science and Technology, Daejeon, 34141 Republic of Korea; 3 University of Science & Technology, Daejeon, Republic of Korea

## Abstract

Silicon probes are multisite electrodes used for the electrophysiological recording of large neuronal ensembles. Optoelectronic probes (OEPs) are recent upgrades that allow, in parallel, the delivery of local optical stimuli. The procedures to use these delicate electrodes for chronic experiments in mice are still underdeveloped and typically assume one-time uses. Here, we developed a micro-drive, a support for OEPs optical fibers, and a hat enclosure, which fabrications consist in fitting and fastening together plastic parts made with 3D printers. Excluding two parts, all components and electrodes are relatively simple to recover after the experiments, via the loosening of screws. To prevent the plugging of OEPs laser sources from altering the stability of recordings, the OEPs fibers can be transiently anchored to the hat via the tightening of screws. We test the stability of recordings in the mouse hippocampus under three different conditions: acute head-fixed, chronic head-fixed, and chronic freely moving. Drift in spike waveforms is significantly smaller in chronic compared to acute conditions, with the plugging/unplugging of head-stage and fiber connectors not affecting much the recording stability. Overall, these tools generate stable recordings of place cell in chronic conditions, and make the recovery and reuse of electrode packages relatively simple.

## Introduction

One important challenge of neuroscience is the understanding of causal links between neural activity and behavior. The last two decades witnessed the development of several new technologies for monitoring the activity of large populations of neurons in behaving animals. Imaging techniques such as two-photon microscopy^1, 2^ and micro-endoscopy^3^ are now commonly used in combination with fluorescent proteins to monitor calcium transients in freely moving or head-restrained animals^4–8^. Such approaches not only allow to monitor unprecedentedly large cell populations (>1000 cells) but also provide direct information about cells spatial organization^8^, morphology and gene expression^6^. In contrast, electrophysiological methods achieve a relatively smaller yield in terms of the number of neurons recorded (hundreds of neurons) but present other benefits^9–15^. They are less invasive when deep brain regions are concerned; they are compact enough to allow simultaneous recordings in different brain regions, and they can resolve signals of single action potentials, *in vivo* (imaging techniques still mainly rely on calcium signals, but see refs 16–18).

High density multi-site electrodes such as tetrodes^10, 11^ and more recently silicon probes^12, 13^ are particularly indicated to monitor large cell populations because the close proximity of recording sites enhance the quality of spike sorting^9–11^. While tetrodes consist of four insulated micro-wires typically bundled together within the labs, silicon probes involve advanced photolithographic processes and therefore higher costs. In counterpart, they present several advantages over tetrodes. First, they are typically designed with a sharp tip to reduce tissue damage during penetration. Second, the recording sites are precisely arranged into geometric patterns that can span over hundreds of microns, allowing one to infer the spatial organization of cells and structures^19–22^. Finally, silicon probes have been the foundation for several lines of hybrid electrodes, such as silicon probes with integrated light guides (‘optoelectronic probe’ [OEP]) or light sources used for optogenetic stimulations^23–28^ and silicon probes with integrated micro-fluidic channels for local drug delivery^29^.

Yet, factors that might deter the use of silicon probes in chronic recordings are the facts that they are expensive and easy to break and that existing methods for chronic implants typically assume a single usage per animal, with no practical options for retrieving the probes at the end of the experiments^30^. And while the potential of hybrid electrodes such as optoelectronic probes (OEP) is conspicuous, their usage in chronic experiments raises an additional set of challenges as they require connection to laser sources. Here, we describe a series of tools for chronic recordings, in either freely moving or head-fixed mice, which facilitate the procedures for silicon probe’s implantation, micro-positioning, and post-experiment recovery. In addition, we present a solution for the use of OEPs in chronic experiments. We provide 3D drawings for all plastic parts we describe (See “Tool availability” in Methods).

## Results

Commercially available silicon probes for chronic recordings are typically coupled to a connector via a flexible cable. While the connector needs to be strongly fixed to the animal’s head in order to sustain the pressure from recording head-stage plugging/unplugging, the silicon probe should be fixed to a micro-drive to allow the tuning of its position. In order to be able to retrieve the silicon probes at the end of the experiments, both the micro-drive and the connector should be easily detachable. To achieve this, we developed an approach based on the principle that recoverable parts are fixed with screws to disposable parts cemented to the animal skull.

We designed several devices, using CAD program (*Solidworks, Dassault Systemes*), which we refer to as (1) the hat, (2) the micro-drive, (3) the OEP support, and (4) the micro-drive handle. The components of the micro-drive and OEP support were printed using a high-resolution UV-curable resin based stereolithography 3D printer (*Asiga/Pico Plus 27*, 27 µm resolution, *PlasClear resin). A*ll other parts were printed using fused deposition modeling technology (FMD, *Stratasys/Mojo*). Miniature screws, nuts and washers were purchased from J.I. Morris Co (Southbridge, MA, USA 01660) or Antrin miniature specialties, INC (Amazon.com).

### Hat

**Figure 1:**
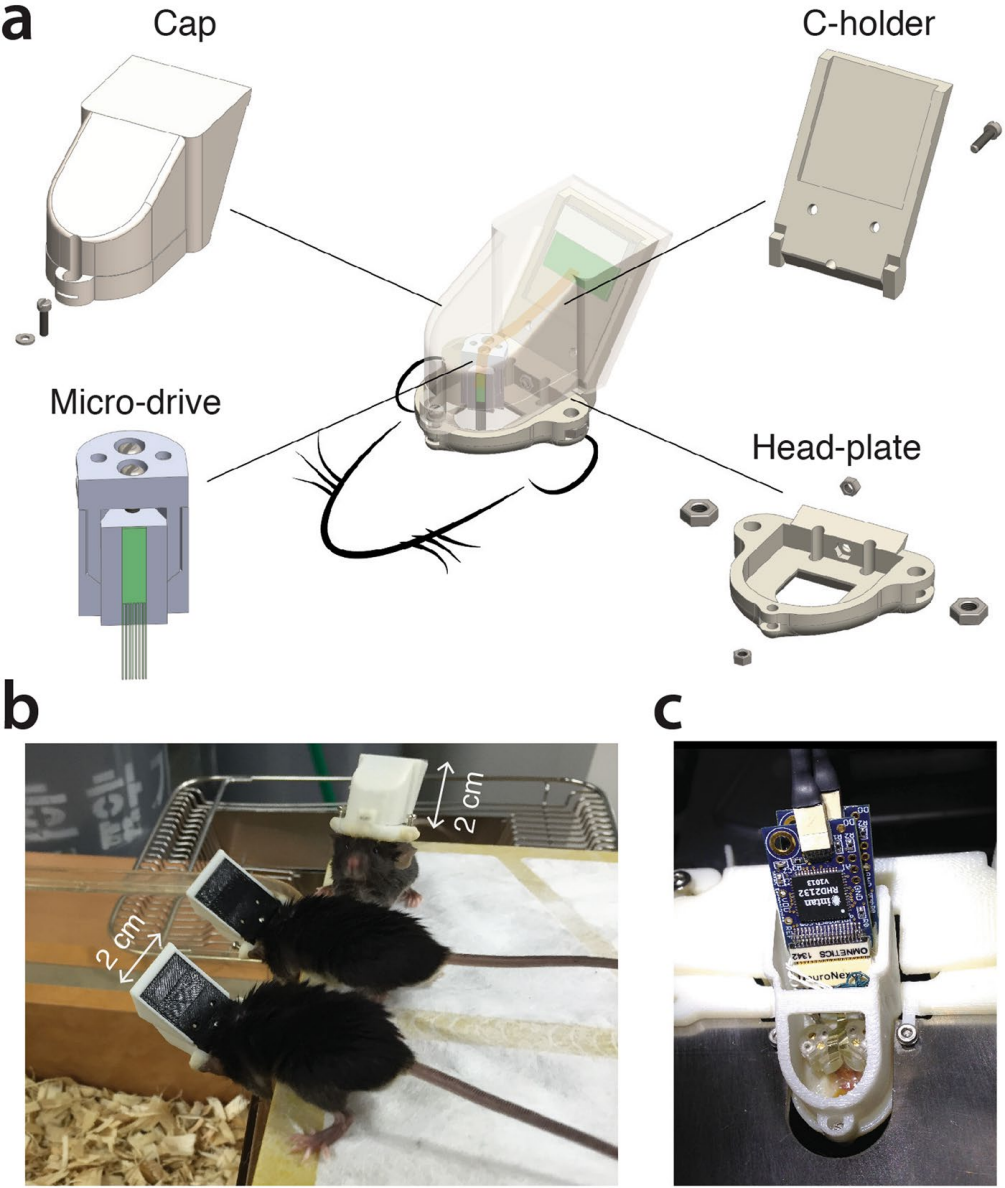
Hat enclosure for chronic recordings, in head-fixed or freely moving mice. (**a**) Micro-drive (lower left), cap (upper left), C-holder (upper right) and head-plate (lower right). (**b**) Mice wearing the hat enclosure. (**c)** Modified cap that allows access to connectors and micro-drives.

The hat enclosure is composed of three parts, referred to as the head-plate, the C-holder (probe connector holder) and the cap (Fig. 1a,b, See Supplementary Information movie). The head-plate is meant to be cemented horizontally on the skull of the mice and present a large opening for accessing the skull in addition to several holes and slots used for head-fixation (side holes, M2), for fixing the C-holder (back hole, 00–90 x ¼ O’Fill) and for fixing the cap (front hole, 00–90 x ¼ O’Fill). Two vertical holes behind the opening are dedicated for passing ground and reference wires. The silicon probe connector is cemented to the C-holder, which fits into the back of the head-plate and is fastened by a screw (00–90 x ¼ O’Fill). The cap is held by a partial embedding of the C-holder and by the tightening of a screw (00–90 x ¼ O’Fill) at the front of the head-plate. Internal edges are designed in the cap to match the C-holder and head-plate contours, ensuring a relatively sealed enclosure. A modified version of the cap, with openings allowing access to connectors and micro-drives (Fig. 1c), is used when performing freely moving recordings. The weights of the head-plate, the C-holder and the cap (including the screws and imbedded stainless-steal nuts and washers) are 1.12, 0.79 and 1.43 grams, respectively.

### Micro-drive

**Figure 2:**
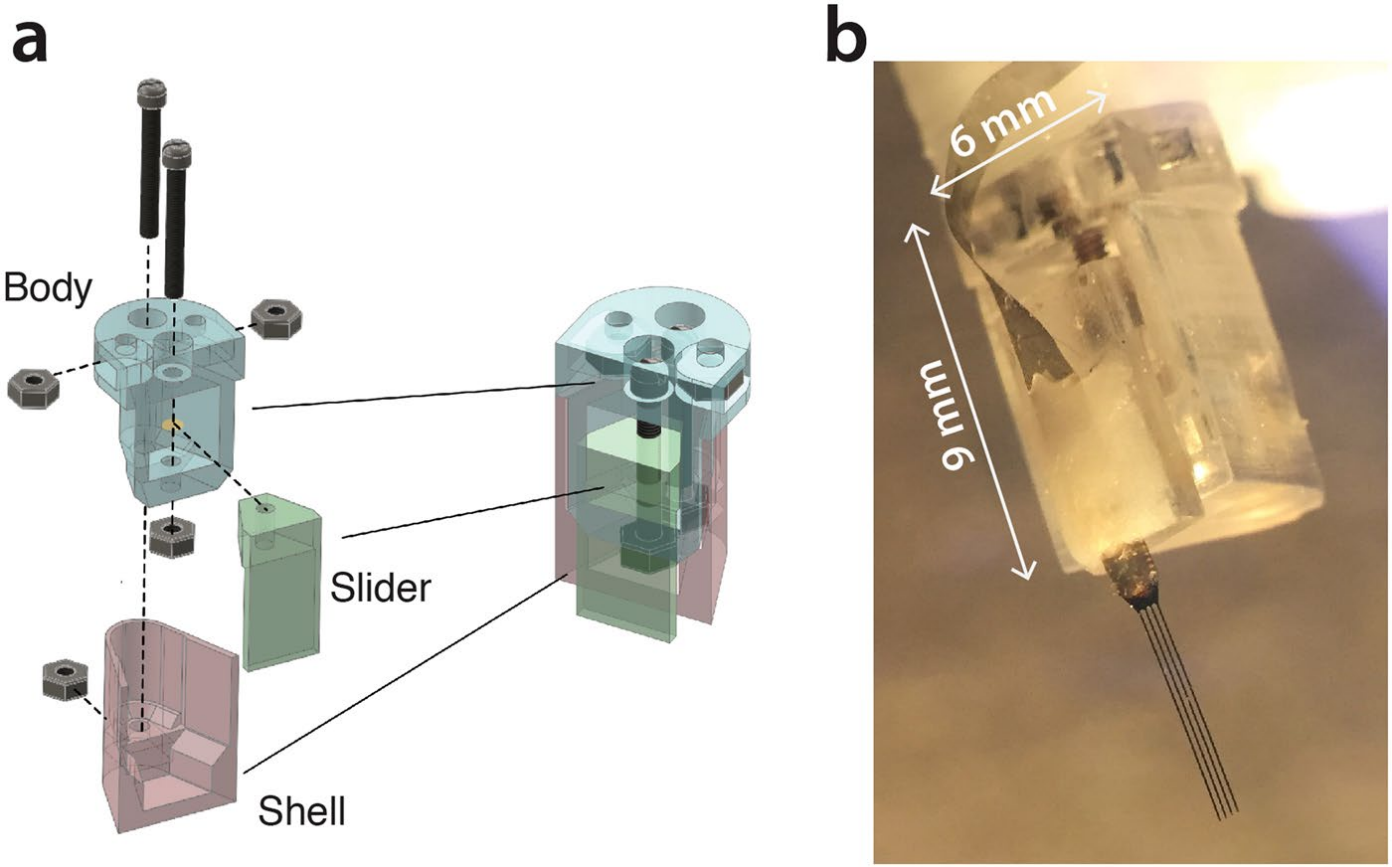
Micro-drive for convenient electrode recovery. (**a**) The slider (green) is held embedded in the body (blue) by the front screw and moves vertically when the screw is turned. The body is held embedded in the shell (red) by the back screw. The shell is the part that is cemented to the animal′s head. The probe and micro-drive can be recovered by unscrewing the body from the shell. A new shell should be printed and attached before the probe is re-used. (**b**) Picture of the micro-drive with a silicon probe cemented to the slider. The micro-drive is fixed to the handle.

The micro-drive is composed of three parts, referred to as the slider, the body and the shell (Fig. 2a, See Supplementary Information movie). The silicon probe is fixed on the slider, which moves linearly relative to the body via the turning of a screw. The body contains a trapezoidal engraving that fits the slider shape and holes for 4 screws to move the slider, to anchor the body to the shell, and to anchor the micro-drive to the micro-drive handle. The shell wraps around the body and is the only part of the micro-drive cemented to the animal skull, such that the electrode package can be recovered by unscrewing the shell.

Under binocular microscope supervision, residuals of print material on the surface and in the holes of the body are removed using respectively a file and a needle (21 gauge). Miniature screws and nuts (000–120 x ¼ O’Fill) are used for assembling the micro-drive. First, the slider is mounted to the body, using a 1/4″ long screw. The hole in the slider is taped during the initial insertion by turning the screw. A nut is fitted to the end of the screw protruding from the bottom of the body and soldered to the screw using acid based flux. Then, three nuts are inserted in designated slots on the body (two nuts) and shell (one nut) and secured using fast-acting cyanoacrylate adhesives. The body is fixed to the shell using a 1/4″ long screw. With the help of micromanipulators and under binocular microscope supervision, a silicon probe is laid against the slider, parallel to the axis of the drive, and fixed to the slider using dental cement.

Altogether, the micro-drive weights 0.4 grams.

### Support for OEP

**Figure 3:**
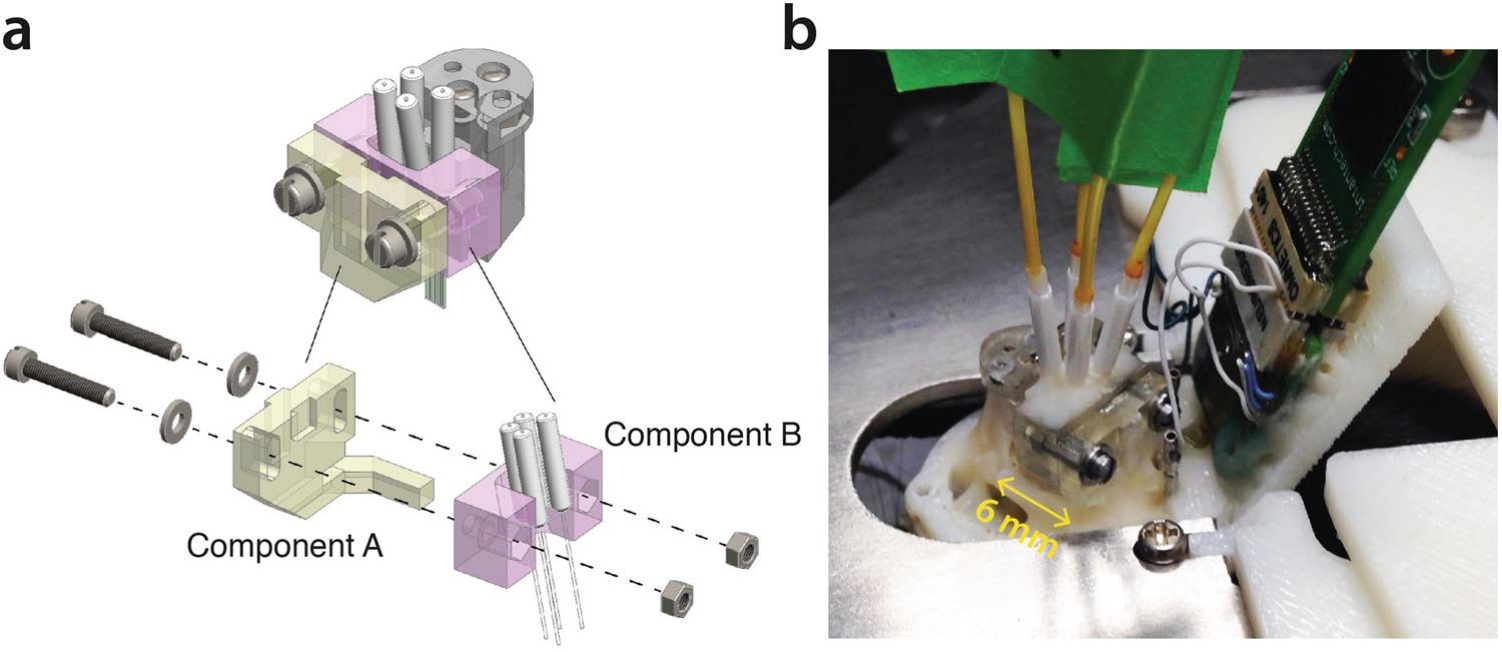
Support for optoelectronic probes. (**a)** Fiber connectors are cemented to part B (pink), which is fastened to part A (yellow) by two screws. The screws are loosened and move freely in the slots of part A when the micro-drive is operated to adjust the electrode position in the brain. The screws are tightened before the optical fibers are connected in order to prevent the plugging pressure from altering the electrode position. (**b**) Picture showing the micro-drive and support for optoelectronic probes cemented to the head-plate. Four optical fibers are plugged to the four connectors of the optoelectronic probe.

Commercial or custom-made OEPs carry light guides that typically need to be connected to lasers during the experiments^23^. The plugging/unplugging of optical fiber connectors involve relatively strong forces, which, if sustained by the slider of the micro-drive, would likely move the electrodes drastically. Therefore, the fiber connectors should be anchored directly to the animal’s skull and not to the micro-drive. On the other hand, the fibers are also tied to the electrodes and need to move in concert when the micro-drive is operated. The support device resolves these two constraints by allowing a switch between two configurations, ‘loose’ versus ‘anchored’ fiber connectors, via the loosening and tightening of two screws (Fig. 3).

The support consists of two components, one that is fixed to the skull (component A) and the other that is fixed to the fiber connectors (component B). Two screws (00–90 x ¼ O’Fill) are used to fasten together the two components. The clearance holes in component A are elongated, allowing the vertical movement of component B when the screws are loosened.

With the help of micromanipulators and under binocular microscope supervision, the support is positioned parallel to the micro-drive, with the optical fiber connectors imbedded in component B. Optical glue (Norland optical adhesive #61) and dental cement are used to fix the optical fiber connectors to the component B, and dental cement is used to bind the component A to the micro-drive shell.

Altogether, the support for OEP weights 0.54 grams.

### Micro-drive handle

**Figure 4:**
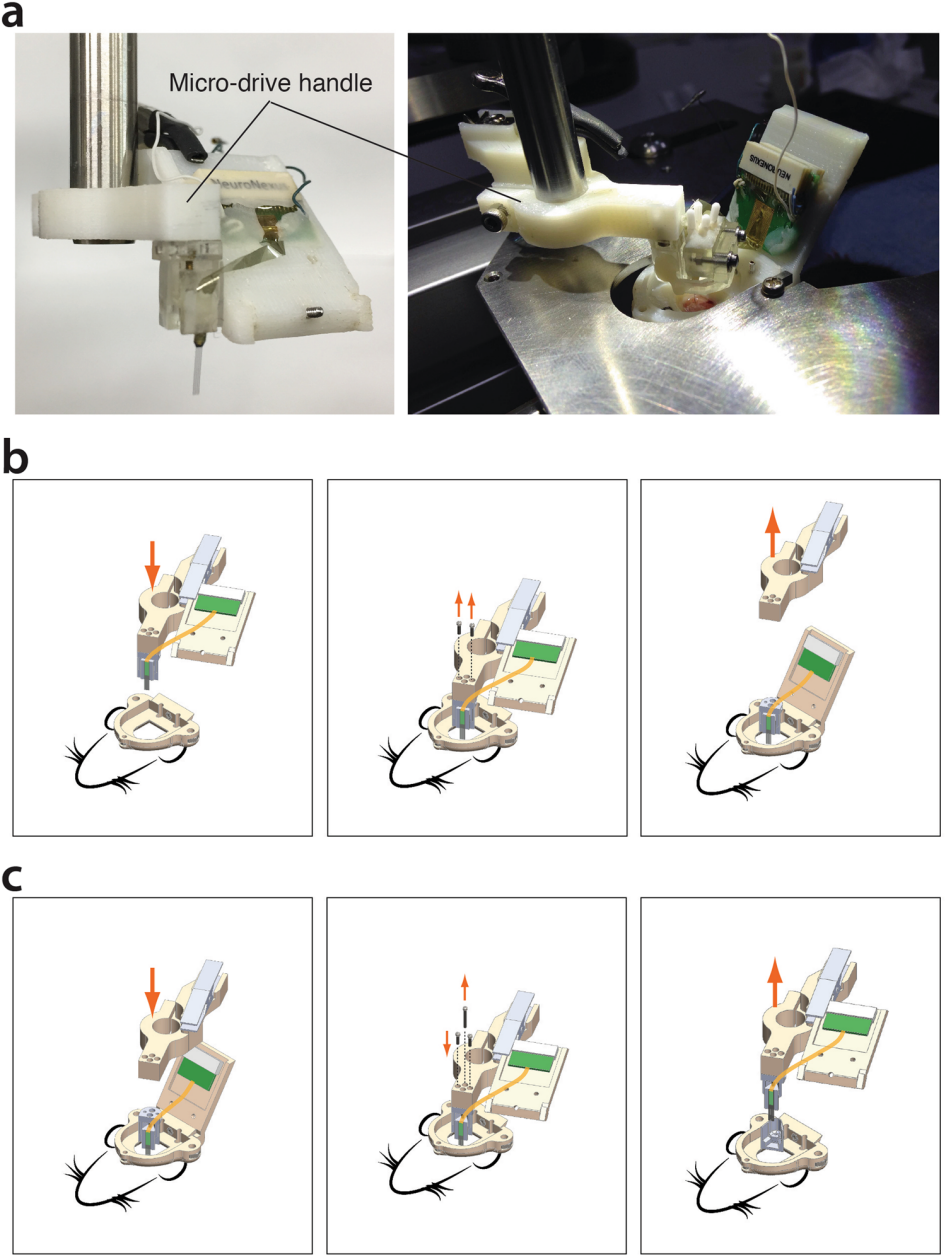
Procedure to implant and recover the electrodes. (**a**) Silicon probe (left) and optoelectronic probe (right) packages ready for implantation. (**b)** Following the probe implant (left) and cementation of the micro-drive to the head-plate, the handle is detached by unscrewing the side screws (centered) and retracted (right). (**c**) To recover the probe after the experiment, the handle is fixed to the micro-drive (center), the back screw holding the micro-drive body to the shell is removed (center), and the electrode/micro-drive assembly is retracted (right).

The micro-drive handle is a simple 3D printed part presenting a hole to fit a stereotaxic post (8 mm diameter), an alligator clip to grab the silicon probe’s connector, and a fixing site for the micro-drive, with two 1/8″ stainless steel screws (000–120) fitting with the micro-drive’s nuts and two holes for allowing access to the micro-drive screws (Fig. 4).

### Surgery procedure for hat and silicon probe implant

During a first surgery under isoflurane anesthesia (supplemented by subcutaneous injections of buprenorphine 0.1 mg/kg), two small watch-screws were driven into the bone above the cerebellum to serve as reference and ground electrodes for the recordings. The head-plate was cemented to the skull using dental acrylic, with ground and reference connectors passing through the assigned holes.

For a period of three weeks, mice were trained to run on a treadmill (head-fixed) and in an open arena. Then a second surgery was performed under isoflurane anesthesia to implant the electrodes. The mouse was installed head-fixed to a stereotaxis device. A small craniotomy was performed with a micro-drill and the *dura mater* removed using a sharp needle. The handle/micro-drive/electrode package was mounted on the rod of a stereotaxic manipulator (Fig. 4a) and moved above the craniotomy under binocular microscope supervision (Fig. 4b). When the tip of the silicon probe was about half a centimeter above the surface of the cortex, the C-holder was detached from the micro-drive handle and fixed to the head-plate. Ground and reference wires from the electrode and the animal were connected together. The recording head-stage (Intan’s RHD2000 acquisition system) was plugged in order to monitor the electrophysiological activity during the probe insertion. The electrode was inserted slowly (~100 µm per min) to the granule cell layer of the dentate gyrus, identified by characteristic features of the local field potential and the emergence of unit activity. The electrode was then retracted 200 µm (the electrode was moved back to the granule cell layer the next day using the micro-drive). Dental cement was applied between the micro-drive shell and the head-plate. When the support for OEP was used, the component A was cemented to the head-plate. After the cement was completely cured, the micro-drive handle was detached from the micro-drive by removing the two dedicated screws. A mixture of bone wax and mineral oil was applied on the craniotomy, and the cap was fixed in place.

It should be mentioned that the overall weight of the hat/micro-drive ensemble (~3.75/~4.25 grams without/with OEP support) did not prevent mice to run in the maze and to climb on the wall/ceiling of their home cage.

### Procedure to retrieve the silicon probe

At the end of the experiment, the mouse was put to sleep under isoflurane anesthesia, and the head-plate was fixed to the stereotaxic device. The micro-drive handle was brought 5 millimeters above the micro-drive using the stereotaxic manipulator (Fig. 4c). The C-holder was detached and grabbed by the alligator clip on the handle. After aligning carefully and lowering the handle until it touched the micro-drive, the two screws holding the micro-drive to the handle were tightened and the screw securing the body to the shell was removed (as well as the two screws holding together the OEP support). The micro-drive/electrode assembly was pulled out slowly using the stereotaxic manipulator. To clean the electrode, a small rotating foam swab controlled by a manipulator was soaked with detergent (Contrad70, Decon Labs, Inc) and was used to brush the recording sites under binocular microscope supervision. The electrode was then rinsed with ultra-pure distilled water and disinfected using alcohol. Following this procedure, the electrodes could be reused multiple times (>6 animal implants) with no apparent decline in signal quality.

### Recordings of hippocampal cell activity

We performed silicon probe recordings in the dentate gyrus using the following three recording settings: (1) acute head-fixed, (2) chronic head-fixed, and 3) chronic freely moving. For both acute and chronic head-fixed recordings, mice ran on a long treadmill belt (2 meters) enriched with visual-tactile cues, previously shown to elicit firing activity similar to place fields in the hippocampus^6, 7, 31, 32^. For the head-fixation, the two side screws of the head-plate were inserted and tightened in slots of a transversal metal plate (2 mm thick) held 3 cm above the treadmill.

The procedure for acute recording in the treadmill was described previously^31^. Briefly, the head-plate was fixed to the mouse skull during a first surgery. Then the mouse was trained to run head-fixed on the treadmill for a period of 3 weeks. The day of the recording, the mouse was put to sleep under isoflurane anesthesia and installed head-fixed in the treadmill apparatus. A craniotomy of ~1 mm^2^ was performed and the *dura mater* was removed. An acute OEP (Buzsaki64sp/Neuronexus silicon probe with 4 out of 6 shanks carrying an optical fiber^23^) was fixed to a stereotaxic micromanipulator and was lowered into the brain at a speed of ~50 μm/min. After the silicon probe reached the target area, the anesthesia was removed. The craniotomy was then sealed with liquid agar (1.5%) applied at near body temperature. Mice typically recovered from anesthesia after 30–45 minutes and then spontaneously started running on the treadmill for water rewards.

Surgery procedures for chronic recordings were performed as described earlier in the section “Surgery procedure for hat and silicon probe implant”, in order to implant a chronic OEP (Buzsaki64sp/Neuronexus silicon probe with omnetic connectors and 4 out of 6 shanks carrying an optical fiber^23^). Chronic head-fixed recordings in the treadmill consisted in installing the mice head-fixed in the treadmill, removing the cap and plugging the recording head-stage and optical fibers. For recordings in the open arena, the modified cap was fixed when small objects were present, to protect the electrodes, but was not essential otherwise.

### Enhanced unit stability in chronic versus acute condition

**Figure 5:**
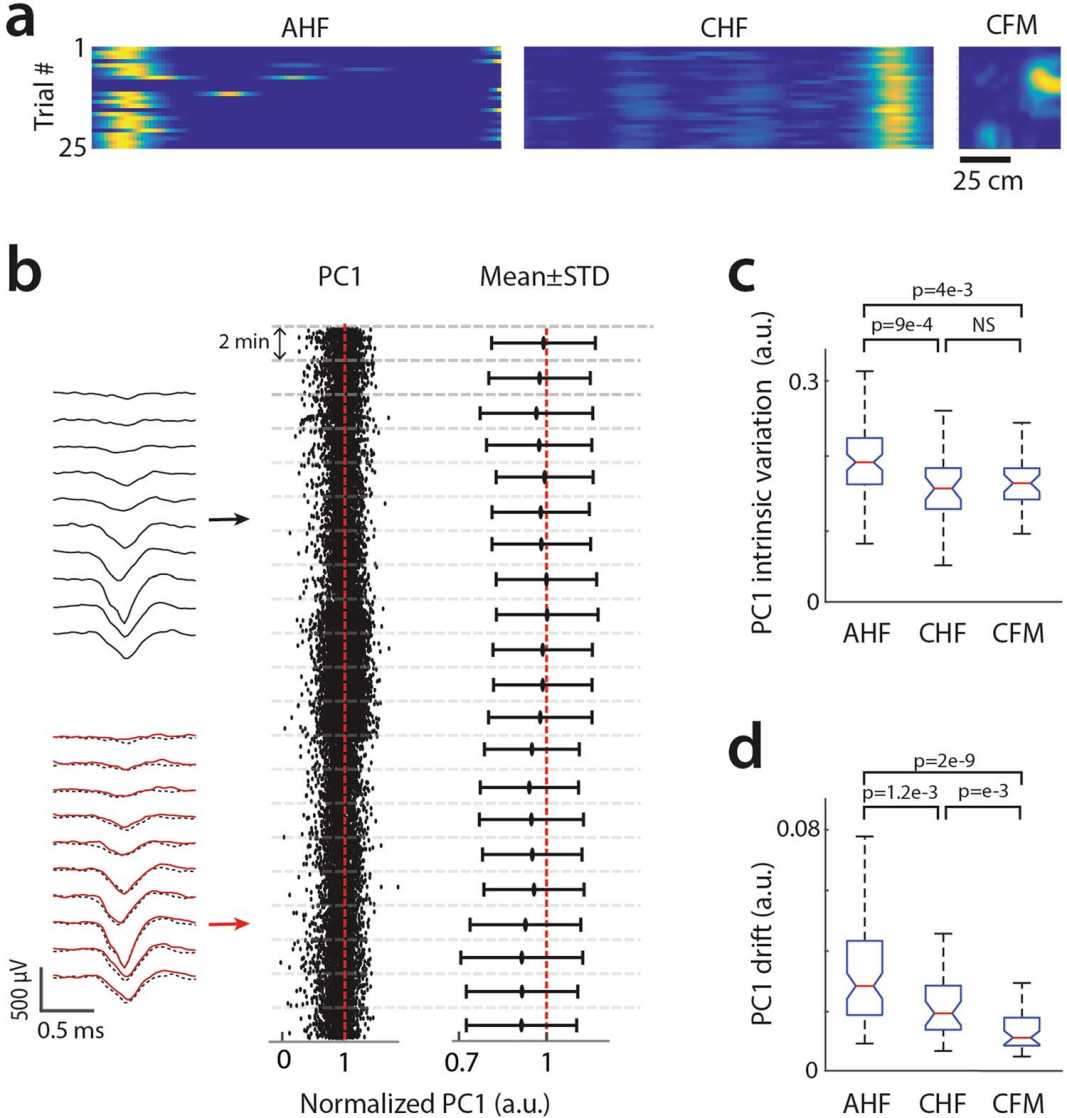
Stability of units for three different recording settings. (**a**) Examples of place cell activity in acute (left, AHF), chronic head-fixed (middle, CHF) and chronic freely moving (right, CFM) settings. (**b**) Temporal evolution of spike waveforms for one cell example. Left, average spike waveforms for 2′ time windows early (black) and late (red) in the recording session. The two waveforms are overlaid (red line and black dash line) to facilitate comparison. Middle, first principal components (PC1) of individual spikes. Right, means ± standard deviations of PC1 for consecutive 2′ windows. PC1 values are normalized by the maximum of the means. (**c**) Population median (red), quartiles (blue) and edges (black) for cells’ intrinsic PC1 variations for the three recording settings. The intrinsic PC1 variation of each cell was defined as the average over all 2′ time windows of the PC1 standard deviations. (**d)** Population median (red), quartiles (blue) and edges (black) for cells’ PC1 drifts for the three recording settings. The PC1 drift of individual cells was defined as the average, over all 2′ time windows, of the shifts (absolute value) in PC1 mean between consecutive windows. (Kruskal-Wallis test; AHF, n = 83 cells; CHF and CFM, n = 33 cells).

Stable place field activity could be observed for all recording conditions tested, that is, acute head-fixed, chronic head-fixed and chronic freely moving (Fig. 5a). However, the stability of spike waveforms varied depending on the settings. To quantify this, we considered cells with a firing rate above 10 Hz (83 cells in 3 acute mice and 33 cells in 3 chronic mice) and performed for each cell a principal component analysis on spike waveforms. We then examined the temporal evolution of the first principal component (PC1) (Fig. 5b). We could identify two distinct forms of PC1 fluctuations, involving short and long time scales. The fast fluctuation was reflected by the spread in PC1 values when considering any time point. As it likely arised from variations in cells/tissue intrinsic properties, it will be referred to as intrinsic PC1 variation. On the other hand, the slower form of variability was characterized by progressive drifts in PC1 values suggestive of electrode movement in the brain and will be referred to as PC1 drift. To quantify each form of fluctuations, we compute the mean and standard deviation of PC1 values for time windows of 2 minutes (2′-PC1-mean and 2′-PC1-std, respectively). For individual cells, the intrinsic PC1 variation was defined as the average 2′-PC1-std over all time windows, while the PC1 drift was defined as the average change (in absolute value) in 2′-PC1-mean between consecutive time windows. In average, both chronic conditions showed lower values than the acute condition for either intrinsic PC1 variation (Fig. 5c) or PC1 drift (Fig. 5d) (for all chronic vs acute comparisons: P < 0.005, Kruskal-Wallis test). Interestingly, chronic freely moving showed slightly lower PC1 drift (but not intrinsic PC1 variation) compared to chronic head-fixed (Fig. 5d) (P = 0.001, Kruskal-Wallis test).

### Impact of plugging/unplugging connectors

**Figure 6:**
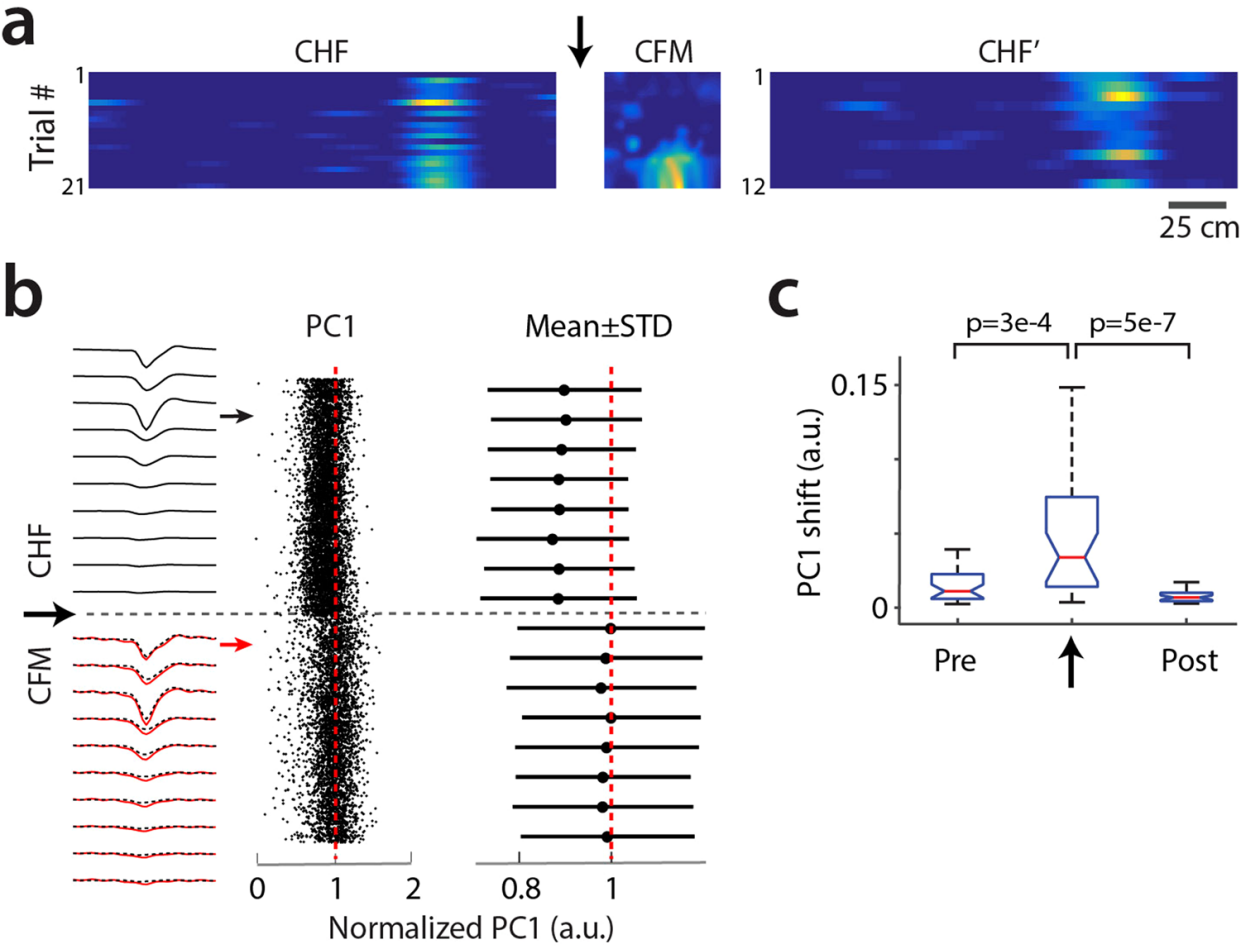
Impact of connector plugging/unplugging on unit stability. (**a)** An example of one cell recorded first in chronic head-fixed setting (CHF), then in chronic freely moving setting (CFM) following the unplugging of optical connectors and the unplugging/plugging of the recording head-stage, and then back in the chronic head-fixed setting (CHF’). The arrow indicates the session transition considered for the analyses shown in b and c. (**b)** Temporal evolution of spike waveforms for one cell example. Left, average spike waveforms for 2′ time windows taken during CHF (black) and CFM (red) recording sessions. The two waveforms are overlaid (red line and black dash line) to facilitate comparison. Middle, first principal component (PC1) of individual spikes. Right, means ± standard deviations of PC1 for consecutive 2′ windows. PC1 values are normalized by the maximum of the means. (**c**) Population median (red), quartiles (blue) and edges (black) for cells’ shifts in PC1 values between the two consecutive 2′ time windows preceding (pre), within (arrow), and following (post) session transition. (Kruskal-Wallis test, n = 33 cells).

Last, we examined the stability of chronic recordings across consecutive sessions in the treadmill (head-fixed) and the open arena (freely-moving), between which the head-stage and optical fibers were unplugged and re-plugged. While place fields from the same neurons could be monitored across consecutive environments (Fig. 6a), spike waveforms were affected by the plugging/unplugging of connectors and/or animal handling between sessions (Fig. 6b), as the change in 2′-PC1-mean was significantly larger at the session transitions than just before or after the transitions (n = 33 cells, p = 3e-4 and 5e-7, respectively, paired t-test) (Fig. 6c). However, for individual cells, the change in 2′-PC1-mean at the transition was considerably lower than the intrinsic PC1 variation (first, second and third quartile: 0.0117, 0.0316, 0.0723 versus 0.1347, 0.1638, 0.1791, n = 33 cells, p = 8.5e-08, paired t-test), indicating that the impact of connector plugging/unplugging was small relative to the intrinsic variability.

## Discussion

We have implemented a set of devices to perform chronic recordings with silicon probes and OEPs in freely moving or head-fixed conditions. While several other versions of micro-drives for chronic recordings have been published or made commercially available^30–35^, our devices are unique in several ways. First, they make possible the recovery of electrodes and connector packages at the end of the experiments. This is a significant advantage considering the cost of silicon probes and the labor required to mount them on micro-drives. Especially in the case of custom made OEPs, the additional work required for fixing optical fibers on the silicon probes’ shanks is particularly challenging^23^. Second, we have developed a mechanism to transiently loosen or fix the OEPs fiber connectors, resolving the paradox that electrode should be movable while connectors should be strongly fixed. Another solution was used in a previous study with chronic OEP implant in rats^23^. The fiber connector was permanently cemented to the animal’s hat, and the fiber segment extending to the probe was devised long enough to allow a curvature. Electrode motion was then possible through the bending/unbending of the fiber. However, because of the limited flexibility of optical fibers, this method required relatively long fibers and therefore large hats, which is not suitable for mice. Other OEP designs avoid the problem by incorporating small light sources directly into the electrode. For one design, laser diodes were juxtaposed to the optical fiber^24^. However, because of the limited power of laser diodes and the absence of lens to collimate the laser, thicker fibers (60 µm diameter instead of 15 µm in ref. 23) had to be used to reach sufficient illumination power, increasing the overall size of electrode and potential tissue damage. The maximum power reached was still less than 100 µW, which is enough for local excitation using channelrhodopsins^23, 36–39^ but limited for less sensitive opsins such as halorhodopsins^23, 40^. More recent OEPs were designed with light-emitting diodes built-in near the recording sites. However, these OEPs are still in developmental stages, with unresolved issues related to power insufficiency, electrical artefact and heat generation^27^. Hence, using an external light-source is still designated for achieving a strong illumination power with minimally invasive light guides. Last, it is noteworthy that our OEP option might not only be useful for OEPs but also for other types of hybrid electrodes, such as silicon probes with integrated micro-fluidic channels^29^.

We observed that spike waveforms were more stable in chronic than acute experiments, and slightly more stable in freely moving than head-fixed conditions. Fluctuations in spike waveforms might be caused by different factors affecting different time scales^41, 42^. For instance, variation in voltage-gated channel activation/inactivation^43^ and blood pulsation-related movements are likely affecting short time scales and might contribute to spike waveform variability within the 2-minute windows we considered. On the other hand, slower changes in spike waveform might reflect drifts of the electrode position in the brain. Our observation that the slow changes were relatively larger in acute recordings is consistent with the fact that the electrodes had less time to stabilize. And the slightly larger variability in head-fixed versus freely moving condition could reflect pressure on the skull generated from the head-fixation.

In conclusion, we have developed a practical set of tools for chronic recordings with silicon probes and optoelectronic probes in freely moving and head-fixed experiments. The tools generated stable recordings and allowed the recovery of electrode packages. The fabrication process involves the 3D printing of plastic parts, which can be either outsourced or implemented within labs with small 3D printers, and then their assemblage using small machine screws and nuts, for which we provided all the necessary information. Given the recent advances in silicon probe and other hybrid electrode development, this set of tools should be useful for a range of experiments in chronic conditions.

## Methods

### Animals

All experiments conformed to the Guide for the Care and Use of Laboratory Animals (NRC 2011). The experimental protocols were approved by the Institutional Animal Care and Use Committee of the Korea Institute of Science and Technology.

6 male C57BL/6 mice between 6 and 7 weeks were used. The mice were housed 2 to 3 per cage, in a vivarium with 12 hours light/dark cycles. Training and recording sessions described next occurred during the light cycles.

### Behavior control and data acquisition

In the treadmill, forward and backward movement were monitored using two pairs of LED and photo-sensors that read patterns on a disc coupled to the treadmill wheel, while the zero position was implemented by a LED and photo-sensor couple detecting a small hole on the belt. From these signals, the mouse position was implemented in real time by an Arduino board (Arduino Uno, arduino.cc), which also controlled the valves for the reward delivery. Position, time and reward information from the Arduino board was sent via USB serial communication to a computer and recorded with custom-made LabView (National Instruments) programs. In the maze, the mice position was monitor using a camera and Noldus Ethovision software (www.noldus.com).

For acute recordings, neurophysiological signals were acquired continuously at 24414 Hz on a 128-channels recording system (Tucker-Davis Technologies, PZ2–128 preamplifier, RZ2 bioamp processor). For chronic recordings, neurophysiological signals were acquired continuously at 30000 Hz on a 250-channels recording system (Intan Technologies, RHD2132 amplifier board with RHD2000 USB Interface Board and custom-made LabView interface).

The wideband signal was digitally high-pass filtered (0.8–5 kHz) offline for spike detection. Spike sorting was performed semi-automatically using custom made programs in Matlab.

### Implementation of the firing rate map of individual neuron

In the treadmill, the length of the belt was divided into 100 pixels. In order to generate a map of firing rates, the number of spikes discharged in each pixel (spike count map) and the time the animal spent in each pixel (occupancy map) was estimated. Spike count and occupancy maps were smoothed by convolving a Gaussian function (15 cm half-height width). The rate map was obtained by dividing the spike count map by the occupancy map.

In the maze, the area of the maze was divided into 50 by 50 pixels, and the rate map was obtained with the same procedure as for the treadmill.

### Statistical analysis

All statistical analyses were performed in Matlab (MathWorks). For each distribution, a Kolmogorov-Smirnov test was used to test the null hypothesis that the sample distribution was derived from a standard normal distribution. If normality was uncertain, we used non-parametric tests as stated in the main text or figures. Otherwise, Student t-tests were used to test the sample mean.

### Tool availability

All CAD drawings (.STL format) for the tools described in the manuscript can be requested directly by email to the corresponding author.

## Electronic supplementary material

Hat assembly movie

Micro-drive assembly movie
